# Preparation of ZnO nanoparticles modified with silane coupling-agents to fabricate anti-UV Poly(vinyl chloride) films

**DOI:** 10.55730/1300-0527.3327

**Published:** 2021-12-23

**Authors:** Xiangmei MA, Zhehao DONG, Bin WANG, Limin LIU, Ruojun YE

**Affiliations:** 1 School of Chemical Engineering, Anhui University of Science and Technology, Huainan, China; 2 Institute of Environment-friendly Materials and Occupational Health of Anhui University of Science and Technology (Wuhu), Wuhu, China; 3 Disaster Prevention and Control in Deep Coal Mines, Anhui University of Science and Technology, Huainan, China

**Keywords:** Anti-UV, ZnO NPs, silane coupling-agent, modified, PVC

## Abstract

The uses of inorganic metal oxide as ultraviolet (UV) absorbers have potential to increase the production of UV protection and can also overcome the disadvantages of organic molecules. In this article, we report an effective technique to fabricating polyvinyl chloride (PVC) films with well UV shielding efficiency. Surface modification of zinc oxide (ZnO) nanoparticles (NPs) with different silane coupling-agents were achieved, and through solution casting technique dispersed within the PVC matrix. Infrared spectroscopy (FT-IR), scanning electron microscopy (SEM), thermogravimetric analysis (TGA) and UV spectrophotometer were applied to study the structures, dispersions, and optical properties. The results showed that the functionalized ZnO NPs could be well dispersed in PVC and endow the polymer composite films with significantly improved anti-UV capability. The facile processing and obtained properties of PVC composites have shown potential for low cost and environmentally sustainable applications in the UV protection field.

## 1. Introduction

The widely used PVC is one type of general-purpose plastic, various advantages are contributing to its widespread use such as low decomposition temperature, economical costs, and ease of synthesis [1–3]. The global consumption of PVC has been expected to be 49.5 million tons in 2020 and is increasing by about 4.9% per year [4]. However, the performance of PVC requires improvement by the incorporation of different additives due to its several defects under different conditions. For example, it suffers from aging and loss of its mechanical properties and appearances under exposure to UV radiation. Anti-UV polymers material that can protect PVC have received significant attention because of the inevitable harmfulness caused by UV light and the deterioration of mechanical properties of PCV [5,6]. With the increasing demand for products that meet the requirement of a comfortable and healthy life, interest in multifunctional materials is increasing correspondingly [7, 8].

During the past decades, many anti-UV agents have been developed as additives to protect PVC from UV degradation and endow PVC with anti-UV efficiency [9–13]. Some organic materials have been utilized as UV blockers, but their limited practical applications owing to phototoxicity, photodegradation and photo-allergenic effects. Inorganic UV blockers are usually environmentally friendly, less toxic, economical, chemically/thermally with a wider UV blocking range and better durability. Several types of metal oxide NPs have been documented such as ZnO, titanium oxide (TiO_2_), and cerium oxide (CeO_2_) [14, 15]. Among them, nano zinc oxide has incredible potential applications, specifically, the utilization of ZnO NPs into polymers has withdrawn the attention of researchers. Advantages of its application include but not limited to low cost, nontoxicity, long-term environmental stability, excellent UV blocking properties and exhibits green-centered fluorescent with UV excitation [16, 17]. Nonetheless, due to the large surface area and high surface energy, nano zinc oxide is easy to aggregate, and PVC suffers from rapid catalytic dehydrochlorination under UV radiation or heat [18]. Many organic supports or coverings contacted with ZnO can be degraded with UV radiation [19, 20]. Thus, the photocatalytic effects of zinc oxide NPs could degrade the polymer matrices [21, 22].

The powerful photocatalytic activity is one of the most important problems which should be seriously considered. Not only that, the properties of the polymer/NPs composites are decided by the dispersion of the NPs, the key aspect is to homogeneously disperse the NPs into the polymer to maintain their desired properties, the ZnO NPs need to be well dispersed in the different base materials. Therefore, the application and development of the polymeric composites have been restricted because of the ZnO poor dispersion, as well as the so-generated deterioration of mechanical properties [23]. Meanwhile, ZnO NPs were incorporated into PVC, the compatibility and dispersion between them would not be ignored [24], and its photocatalytic activity must be reduced to make them safer UV absorbers. For solving the mentioned problems, a lot of new synthetic methods have been developed to prevent the particles from agglomeration and increase the dispersions [25, 26]. Researchers have focused on the synthesis of new hybrid materials such as layered double hydroxide (LDH)/polymers, polymer/inorganic, and organic/inorganic [27].

Surface modification of ZnO is an effective method to improve its UV blocking effect, compatibility with polymer, and reduced photocatalytic activity. Many experiments have proved that a rational design of functional groups with different properties can lead to different application properties [28–30]. The related researches have confirmed that the utilization of nano zinc oxide is an efficient approach for enhancing the UV blocking properties of polymers such as polylactide [31], poxy resin [32] and poly(methyl methacrylate) [33]. Therefore, the ZnO NPs can be surface modified for fewer dehydrochlorination and a better dispersion [34].

Silane coupling-agents (SCA) are widely used as surface modifiers of NPs, for their lower toxicity and refractive index, easy-controllable reaction and so on [35]. Researches have shown that the amounts and types of silane coupling-agents influence the properties of NPs such as the mechanical, optical, processability and barrier properties etc. [36]. However, the study of SCA-based ZnO hybrid in relation to UV protection applications is still in its infancy. The effects on the anti-UV properties and catalytic activity of PVC by adding surface functionalized ZnO NPs with different SCA are rarely reported.

Therefore, in this article, we made an attempt to produce efficient UV-shielding films using PVC as base material and SCA-functionalized ZnO as additives [37]. First, surface functionalization of ZnO with TEOS to obtain ZnO@SiO_2_ NPs, further the outer shell of the ZnO@SiO_2_ was modified with APTES in order to get more Si-O bonds and oil-soluble propylamino groups. The obtained product was mixed with PVC and taken as film forming material. The effect of the SCA functionalized ZnO on the dispersion in PVC, anti-UV properties and catalytic activities for PVC decomposition were investigated. It was found that the functionalized ZnO as a good UV absorber exhibited excellent properties such as dispersed stability, better anti-UV properties and less catalytic activity compared to ZnO NPs. Furthermore, this research indicated the promising potential of the ZnO-SCA hybrid in the cosmetic industry.

## 2. Experimental

### 2.1. Materials

PVC particles (SG5, the average polymerization degree about 1000) and TiO_2_ were purchased from Xinkaiyuan Chemical Co. (China) Ltd. Rhodamine B (RhB) and ZnO NPs (30 ± 10 nm) of analytical-grade were supplied by Aladdin (Shanghai Co., Ltd., China). All other reagents of analytical grade were purchased from Qiangsheng Chemical Reagent Co., Ltd. (China) and used without purification.

### 2.2 Synthesis of the highly dispersed anti-UV agents

The surface functionalization of ZnO NPs was carried out (illustrated in Scheme) using silane coupling-agents (TEOS and APTES).

#### 2.2.1 Functionalization of the ZnO NPs

ZnO NPs (3 g) were placed in 120 mL ethanol and followed by sonicated for 1 h. After that, ammonia solution (3.4 mL) was added to the solution with pH≈10. Separately, TEOS (10 mL) was added ethanol (10 mL) dispersed using sonicate, and drop-wise to the mixture of ZnO and ammonia solution. The reaction was kept at 60 °C for 16 h under the condition of continuous stirring. The precipitate was centrifuged, washed with 30% ethanol aqueous solution several times. The product (denoted as ZnO@SiO_2_) was dried in an electrothermal drying oven.

#### 2.2.2 Functionalization of the ZnO@SiO_2_ NPs

The functionalization of ZnO@SiO_2_ was modified with APTES [38]. Briefly, ZnO@SiO_2_ (0.8 g), methanol (100 mL), and water (100 mL) were placed in a 500 mL round-bottom flask and sonicated for 1 h, then APTES (8 mL) and methanol (8 mL) were added to the ZnO@SiO_2_ methanol/water mixture, the mixture was stirred at 40 °C for 16 h, was centrifuged, the product (denoted as ZnO@SiO_2_-g-APTES) was washed with 30% ethanol and was dried at 60 °C.

### 2.3 Preparation of PVC/NPs composite films

PVC/NPs composite films were prepared by the casting method. PVC (1.0 g) was dissolved in N,N-dimethylformamide (DMF, 10 mL) to form a homogeneous solution. NPs were ultrasonically dispersed in DMF (10 mL) and were added into the above mentioned PVC solution, were stirred for 2 h and were ultrasonically dispersed 0.5 h to form a homogeneous solution. Finally, they were casted onto a glass plate and were dried in a vacuum oven at 70 °C for 24 h. The corresponding composite films were denoted respectively. For comparison, PVC film was also prepared by the same way. All the obtained composite films were about 50 μm.

### 2.4. Characterization

FT-IR was performed by the Bruker VECTOR-22 FT-IR spectrometer, the samples were ground with KBr and compressed into a pellet. SEM was performed with the SU8020 microscope, the samples were coated with a thin layer of gold palladium for analysis.

UV-vis absorption spectra were recorded by the Shimadzu UV-2700 at room temperature.

Thermogravimetric analysis (TGA) was carried out by the Hitachi Diamond instrument, samples around 10 mg at a heating rate of 10 °C/min in a temperature range of 30–600 °C under a nitrogen atmosphere.

The anti-UV ability of the prepared film was evaluated under UV light with a wavelength of 365 nm (20W) by the degradation behavior of RhB with TiO_2_ as a photocatalyst. Prior to irradiation, 50 mL of 10^−5^ M RhB solution containing 50 mg TiO_2_ was ultrasonically dispersed for 10 min, and the suspension was stirred for 30 min in the dark to reach adsorption-desorption equilibrium. Then, the beaker was covered with PVC or PVC/NPs composite films, irradiated under 1.66 w/m^2^ UV light (wavelength = 365 nm). The degradation process was carried out with constant stirring. At the given time, 6 mL of suspension was collected, and centrifuged to remove the solid, the supernatant concentration of RhB was measured by the UV absorbance at 553 nm. All should be back into the reaction system after the test was over. The relative content (I) of rhodamine B after irradiation is calculated using the following formula:


$$\text{I}=({\text{A}}_{\text{t}}/{\text{A}}_{0})\times 100\%$$

where A_0_ and A_t_ represent the UV absorption values of rhodamine B solution irradiated by UV light for 0 and t min, respectively. The results were used to assess the UV-shielding performance of nanoparticles.

## 3. Results and discussion

### 3.1. FTIR analysis

**Figure 1:**
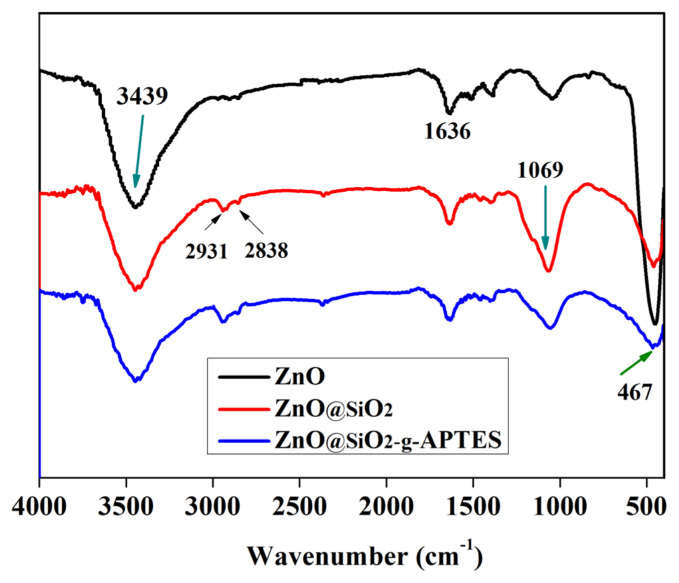
FT-IR spectra of NPs.

The FT-IR spectra of NPs were recorded and depicted in Figure 1. The data affirmed the formation of a chemical bond between SiO_2_ and ZnO. The peak positions of the spectrum were mainly the same, the peak at 467 cm^−1^ is Zn-O bond, the peak (3439 cm^−1^) appearing in the range 3675–3031 cm^−1^ is attributed to the stretching vibration of the -OH groups or the symmetric stretching and overlapping of the N-H bond [39]. While the peak at 2931 cm^−1^ represents the asymmetrical C-H stretching of alkyl groups [40], the peak at 1636 cm^−1^ is attributed to the absorption of water [41]. The peak at 1069 cm^−1^ is attributed to Si-O bond asymmetric stretching, respectively. Hence, the results declared that the ZnO was successfully functionalized with silane coupling-agents.

### 3.2. SEM analysis

**Figure 2:**
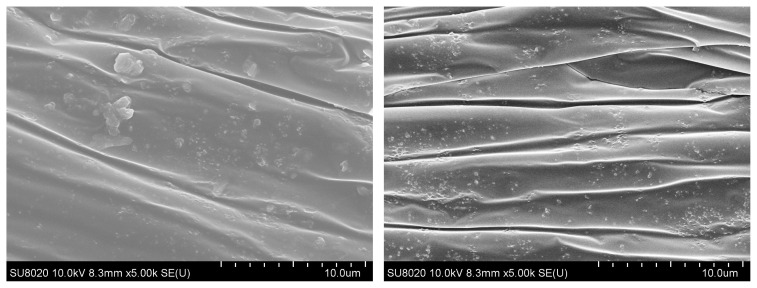
SEM photograph s of the PVC/ZnO@SiO_2_ (2%) and PVC/ZnO@SiO_2_-g-APTES (2%) films.

Dispersion of NPs into polymers plays a major role in critical applications. Polymer nanocomposites were prepared through solution blending of PVC and NPs in DMF followed by casting thin films. Figure 2 shows the dispersion of representative films containing NPs. Nanocomposites with ZnO@SiO_2_ showed some aggregation, visible as areas of white patches in Figure 2a. In contrast, films prepared with ZnO@SiO_2_-g-APTES (Figure 2b) displayed no visible aggregates. The ZnO@SiO_2_-g-APTES NPs are lower than that of the ZnO@SiO_2_ aggregates due to the grafted APTES molecules. According to the literature [42], the higher dispersion of the ZnO@SiO_2_-g-APTES NPs is ascribed to the “softer” APTES shell outside of the nanohybrids, the double-shelled ZnO@SiO_2_-g-APTES NPs have a more homogeneous dispersion than ZnO@SiO_2_ NPs because of the interfacial stereocomplexation. Consequently, the SEM images support the result that ZnO@SiO_2_-g-APTES disperse far better than ZnO@SiO_2_ in PVC. According to the above discussions, ZnO@SiO_2_-g-APTES was well dispersed in PVC composite film.

### 3.3. Thermal stability properties

**Figure 3:**
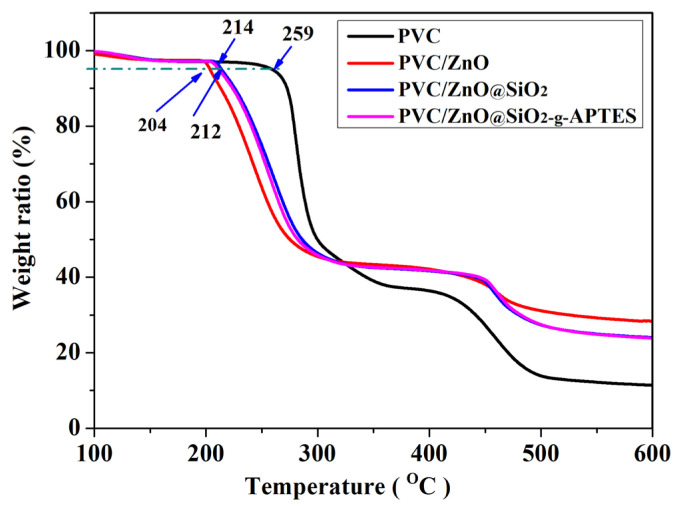
TGA curves of PVC films.

The thermal stability of polymer is an important factor restricting its real-world applications and melt-processing. Furthermore, TGA measurements were used to characterize thermal stability of the PVC/NPs composite films. Typically, the pyrolysis of PVC mainly has two stages process: the release of HCl and degradation of the polymer chain [43]. Figure 3 provides the comparison of the mass loss degradation of the PVC/ZnO@SiO_2_-g-APTES (2%) and PVC/ZnO@SiO_2_ (2%) films by using pure PVC film as a reference in a nitrogen atmosphere from 30 °C to 600 °C. The weight loss of less than 5% can be attributed to the loss of remaining solvents and water. The onset thermal decomposition temperature for 5% weight loss (T_0.05_) of PVC, PVC/ZnO (2%), PVC/ZnO@SiO_2_ (2%) and PVC/ ZnO@SiO_2_-g-APTES (2%) were 259, 204, 214, and 212 °C, respectively. The values (T_0.05_) of PVC containing ZnO@SiO_2_ and ZnO@SiO_2_-g-APTES have no obvious difference, but both are significantly higher than PVC containing ZnO. This phenomenon can be attributed to the assumption: (1) ZnO NPs catalyzed the PVC decomposition to release hydrogen chloride, (2) ZnO NPs absorb more heat and transfer it to the PVC matrix and causing early degradation, or it may be ZnO intermolecular weak forces [44].

### 3.4. Anti-UV properties of films

**Figure 4:**
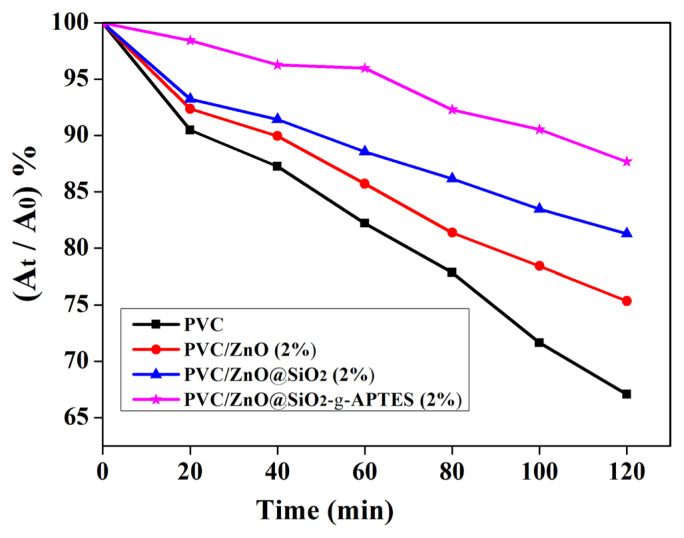
The photodegradation results of RhB solutions protected by PVC composite films with different NPs.

The prepared pure PVC and PVC/ZnO composite films were carried out to quantify the anti-UV capability by photocatalytic degradation of RhB solution using TiO_2_ as the photocatalyst. As shown in Figure 4, for pure PVC, PVC/ZnO (2%), PVC/ZnO@SiO_2_ (2%) and PVC/ZnO@SiO_2_-g-APTES (2%) films, the RhB solution was degraded values of 67, 75, 81, and 88% respectively when UV irradiation for 120 min, which indicates the excellent anti-UV efficiency of the composite film. The anti-UV ability of the PVC/ZnO@SiO_2_-g-APTES nanocomposite is much higher than those of the PVC/ZnO (2%), pure PVC, and PVC/ZnO@SiO_2_ nanocomposite with the same NPs content. The better anti-UV ability performance of the PVC/ZnO@SiO_2_-g-APTES nanocomposite is also due to the fine and uniform dispersion of ZnO@SiO_2_-g-APTES nanohybrids which generates larger specific surface areas [42]. In addition, compared to that of the original RhB solution, the solution protected by the PVC/ZnO@SiO_2_-g-APTES film exhibits negligible discoloration, whereas the solution protected by pure PVC exhibits an obvious discoloration. The results indicate that the as-prepared PVC/ZnO@SiO_2_-g-APTES films possess excellent anti-UV performance, that is, ZnO@SiO_2_-g-APTES can work as a UV-shielding agent for the PVC-based film, and similar results have been reported in many other inorganic-organic composites [45,46].

**Figure 5:**
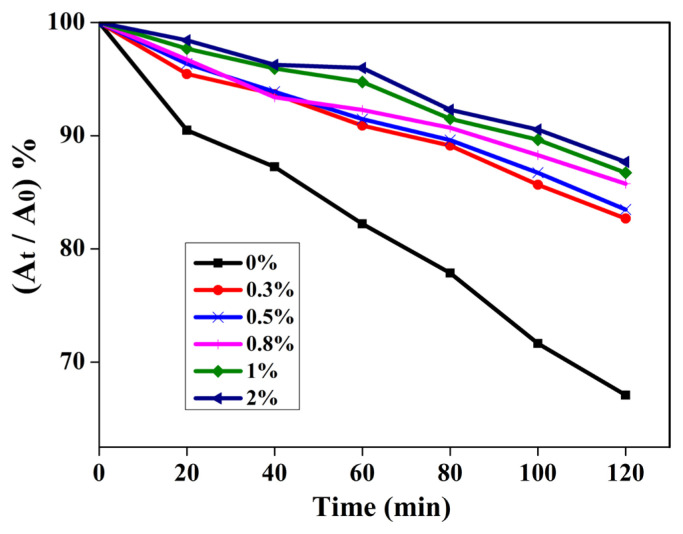
The photodegradation results of RhB solutions protected by PVC/ZnO@SiO_2_-g-APTES. composite films.

The influence of the different weight ratios of NPs on the anti-UV capability of PVC/ZnO@SiO_2_-g-APTES films was also investigated representatively. As shown in Figure 5, with increasing content of NPs from 0% to 2% were used as the protecting film, the RhB solution has an obvious degradation rate and values from 67% to 88%. These results also indicated that the content of ZnO@SiO_2_-g-APTES is a significant factor in the enhancement of the anti-UV performance, and it could absorb energy from UV light and then emit as heat or electromagnetic radiation [47]. That is, the ZnO@SiO_2_-g-APTES can be used as an ideal additive to enhance anti-UV capability of the PVC films.

## 4. Conclusion

In summary, silane coupling-agents (TEOS and APTES) were chosen as the surface modifier and covalently coated on the ZnO NPs through the surface modification process. The obtained double shell structured NPs were used as anti-UV additives to prepare PVC composite films by casting way. The SiO_2_ shell has not only excellent anti-UV performance, but can prevent chemical reactions between the ZnO NPs and the APTES, the APTES shell can also easily interact with the PVC and facilitates the uniform dispersion of ZnO@SiO_2_. The composite film exhibits higher anti-UV efficiency and well NPs dispersibility and are expected to have great practical applications in various different types of materials.

## Figures and Tables

**Scheme:**
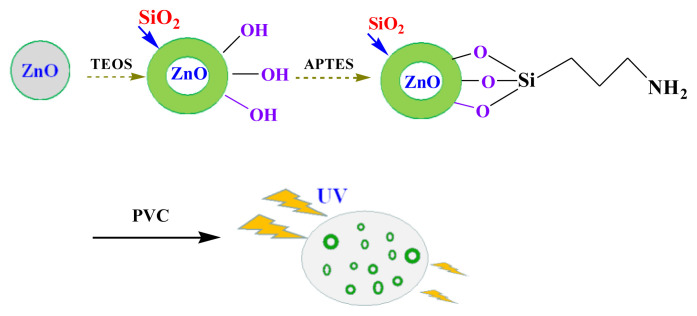
Synthetic route of the highly dispersed anti-UV agents.
